# Single-Blinded Study Highlighting the Differences between the Small Intestines of Neonatal and Weaned Piglets

**DOI:** 10.3390/ani11020271

**Published:** 2021-01-21

**Authors:** Chen Yuan, Penghao Zhang, Yuxin Jin, Abid Ullah Shah, En Zhang, Qian Yang

**Affiliations:** MOE Joint International Research Laboratory of Animal Health and Food Safety, College of Veterinary Medicine, Nanjing Agricultural University, Weigang 1, Nanjing 210095, China; yuanchen060624@163.com (C.Y.); zphforever@163.com (P.Z.); jyx843729845@163.com (Y.J.); abidullahshah@yahoo.com (A.U.S.); MrJohn1994@163.com (E.Z.)

**Keywords:** neonatal piglets, weaned pigs, small intestine, pattern recognition receptors, immune cells

## Abstract

**Simple Summary:**

The gut mucosa of pigs, which contains intestinal epithelium and subepithelial immune cells, forms a barrier against microorganisms. Nonetheless, infectious diseases of the digestive tract remain the most frequent and recurrent conditions in the swine industry. Changes in intestinal morphology and structure primarily occur at birth and during weaning. However, the difference in the intestinal structures between neonatal and weaned piglets remains unclear. In this study, for the first time, we evaluated the differences in the small intestine between neonatal (0-day-old) and weaned piglets (21-day-old) and analyzed the morphology and immunological components of the small intestines of 0- and 21-day-old piglets, thereby providing preliminary data for future mechanistic studies.

**Abstract:**

The gut is one of the body’s major immune structures, and the gut mucosa, which contains intestinal epithelium and subepithelial immune cells, is the primary site for eliciting local immune responses to foreign antigens. Intestinal immune system development in pigs is a transitional period during birth and weaning. This study compares the morphological and immunological differences in the small intestine of neonatal and weaned piglets to potentially prevent intestinal infectious diseases in neonatal piglets. Histological analyses of weaned piglet intestines showed increased crypt depth, higher IEL count, and larger ileal Peyer’s patches compared with those of neonates. Additionally, the ileal villi of weaned piglets were longer than those of neonatal piglets, and claudin-3 protein expression was significantly higher in weaned than in neonatal piglets. The numbers of CD3^+^ T, goblet, and secretory cells were also higher in the small intestines of weaned piglets than in those of neonates. No significant differences were observed in the secretory IgA-positive cell number in the jejunum of weaned and neonatal piglets. The mRNA expression of most pattern recognition receptors genes in the duodenum and jejunum was higher in the weaned than neonatal piglets; however, the opposite was true in the ileum. The mRNA levels of IL-1β and TNF-α in the jejunal and ileal mucosa were higher in weaned piglets than in neonatal piglets. There were significantly fewer CD3^+^, CD4^+^, and CD8^+^ T cells from peripheral blood-mononuclear cells in neonatal piglets. Our study provides insights regarding the different immune mechanisms within the small intestines of 0- and 21-day-old piglets. Studies on the additional developmental stages and how differences in the small intestines affect the response of pigs to pathogens remain warranted.

## 1. Introduction

The gut significantly influences the maintenance of good health and production of pigs [1,2]. The small intestine is divided into three sections namely duodenum, jejunum, and ileum. Besides digestion and absorption of nutrients, intestinal mucosa in pigs plays an important role in combating foreign antigens, including natural toxins and pathogenic and commensal microorganisms [3,4]. Hence, the gut is a major immune organ, and the gut mucosa is the primary site for eliciting and mediating local immune responses [3,5]. Nonetheless, infectious diseases of the digestive tract remain the most frequent and recurrent conditions in the swine industry. It has been reported that various viruses, such as coronavirus transmissible gastroenteritis virus (TGEV), porcine epidemic diarrhea virus (PEDV), and rotavirus A (RVA), primarily infect neonatal piglets [6,7,8,9], but the underlying mechanism is not yet fully understood. Infections with these viruses, and other pathogens, can lower the feed conversion efficiency by inducing diarrhea thereby posing major challenges to the swine industry.

There are two major transitional periods during the development of the intestinal immune system in pig; one at birth and the other at weaning [10,11]. The porcine intestinal immune system is immature at birth [12], and thus, piglets are most susceptible to pathogenic organisms during the neonatal stage [13]. Today, the weaning age of pigs has declined [14], and it has been reported that weaning of Wuzhishan piglets at 21 days of age may have positive effects on the adaptive immune system [15]. Previous studies have investigated the quantitative changes in the two main stem cell populations in the small intestines of 0- and 21-day-old piglets [16]; however, differences in morphology and immunological components of small intestines between neonatal and weaned piglets remain unknown. Therefore, in this study, we compared the physical and immunological differences of the small intestines between neonatal (0 day) and weaned piglets (21 days) to provide preliminary data for future mechanistic and developmental studies.

Pigs are one of the most economically important livestock and are true omnivores sharing similar anatomy and physiology of the digestive system to that of humans, making them a suitable animal model to investigate human intestinal diseases and the biological pathways underlying mucosal functions and development [17,18,19]. Therefore, the use of pigs as an animal model can aid in revealing the structure and function of the intestine in humans.

## 2. Materials and Methods

### 2.1. Animals

Six 21-day-old (weaners; weighing 8–10 kg) and six 0-day-old (neonates; weighing 1.10–1.30 kg)—male cross-bred Duroc/Landrace/Yorkshire piglets were obtained from the Jiangsu Huai’an Pig Farm (Huai’an, China). The piglets were randomly selected and housed indoors at the Jiangsu Huai’an Pig Farm, under constant conditions of 60% humidity, 26 °C, and 12 h light/dark cycle as well as free access to food and water. Our study was based on age-related difference of pig’s small intestine from the same family. All experimental procedures were approved by the Institutional Animal Care and Use Committee of Nanjing Agricultural University, and the National Institutes of Health guidelines for the performance of animal experiments were followed.

### 2.2. Material Collection and Preparation

Each piglet (Jiangsu Huai’an Pig Farm, Nanjing, China) was euthanized by an intravenous injection of pentobarbital sodium (100 mg/kg) before sample collection. To determine the complete anesthetization of the piglets before opening the abdomen, their palpebral and withdrawal reflexes were checked. Duodenum, jejunum, and ileum tissue samples were collected from the piglets and fixed in 4% paraformaldehyde for 48 h at 25 °C. After fixation, the samples were sectioned into small pieces. Then the small pieces (1 cm) dehydrated using a graded ethanol series (75, 85, 95, absolute alcohol and absolute alcohol, each for 1 min in that order). The dehydrated blocks were embedded in paraffin wax and kept at room temperature to dry. Next, the tissue samples were sliced longitudinally into 5-μm-thick sections using a microtome. The sections were dried horizontally on a warming tray (37 °C) overnight, dewaxed in xylene, rehydrated in a graded series of ethanol absolute alcohol, 90, 80, and 70%, each for 1 min in that order, and washed in phosphate-buffered saline (PBS). These tissue sections were then used in the subsequent experimental analyses. All observations were single-blinded. In addition, small intestine tissue, including duodenum, jejunum, and ileum (50–100 mg) was placed in 1.5-mL cryogenic vials containing 1 mL RNAiso Plus (Thermo Fisher Scientific, Inc., Waltham, MN, USA) and triturated in a Tissuelyser-24 (Shanghai Jingxin Industrial Development Co., Ltd., Shanghai, China). The triturated tissue was then used in the subsequent RNA isolation. Whole blood from piglets was collected with a syringe containing an anticoagulant. The obtained peripheral blood-mononuclear cells (PBMCs) were used for subsequent flow cytometry. The above samples were repeated three times per piglet and were collected at different points on the same day.

### 2.3. Histological Analysis

The prepared tissue sections were stained with hematoxylin and eosin (H&E) and examined using light microscopy (BH-2, Olympus, Tokyo, Japan). The methods of H&E staining have been previously reported [20,21]. The H&E staining of the sections was conducted using hematoxylin (0.5% for 20 s) and eosin (0.5% for 5 s) at room temperature. After staining, the sections were dewaxed in xylene and dehydrated through increasing concentrations of ethanol (75, 85, 95% and absolute alcohol, each for 1 min in that order) and xylene (10 min) at room temperature, and finally sealed with a coverslip. The size of the ileal Peyer’s patches (PPs), villus height, and crypt depth were measured using computer-assisted morphometry (Image-Pro Plus software, Rockville, MD, USA).

### 2.4. Immunohistochemistry

Antigens were recovered from the tissue sections by placing them in a citrate buffer (pH 6; 90–95 °C) for 15 min. Then, the sections were treated with 0.3% hydrogen peroxide at room temperature for 15 min and washed with PBS to quench endogenous peroxidase activity. To avoid the non-specific binding of antibodies, the sections were blocked using 5% bovine serum albumin for 30 min at room temperature. After incubating with the primary antibodies overnight at 4 °C, the sections were treated with biotinylated secondary antibodies for 1 h at room temperature (Table 1). To visualize the immuno-positive cells, the sections were stained with diaminobenzidine (DAB) for 60 min at room temperature and sealed with neutral balata. The respective isotypes were used as negative controls. The sections were visualized using a light microscope (Olympus CX23; Olympus Corporation, Tokyo, Japan) at a magnification of 400× or 100×. Different fields (400× or 100×, *n* = 10) of each tissue in each piglet were counted for the statistical analysis.

### 2.5. Immunofluorescence

The tissue sections were incubated with 0.4% Triton X-100 in PBS for 5 min. After blocking with 5% bovine serum albumin in PBS for 1 h, the sections were stained with *Ulex europaeous agglutinin-1* (UEA-1) antibodies at room temperature for 2 h. PBS was used instead of the antibodies in control samples. After staining with 4′,6-diamidino-2-phenylindole (DAPI), the sections were observed using confocal laser scanning microscopy (LSM-710; Zeiss, Oberkochen, Germany).

### 2.6. RNA Isolation and Real-Time Quantitative PCR

Total RNA was extracted from the tissues using a TRIzol^®^ Plus RNA Purification kit (Thermo Fisher Scientific, Inc.). One microgram of total purified RNA was reverse transcribed to cDNA, using the PrimeScript™ RT-PCR kit (Takara Biotechnology Co., Ltd., Dalian, China), as follows: one cycle of 37 °C for 15 min followed by 85 °C for 5 s. RT-qPCR analysis was performed with 2 μL of diluted cDNA (vol:vol, 1:5) to perform RT-qPCR analysis, by ABI 7500 PCR system (Life Technologies; Thermo Fisher Scientific, Inc.) and SYBR-Green qPCR Master Mix (Takara Biotechnology Co., Ltd., Dalian, China) according to the manufacturers’ protocols. The thermocycler reaction involved a pre-incubation period of 95 °C for 30 s, followed by 40 cycles of 95 °C for 5 s and 60 °C for 31 s. Specific primers referred from the studies by Zong et al. (2019), Temeeyasen et al. (2017), Xia et al. (2018), and Wang et al. (2020) are shown in Table 2 [22,23,24,25]. Data were normalized against GAPDH mRNA levels and are expressed as fold differences between the neonatal and weaned piglets, calculated using the 2^−ΔΔCT^ method.

### 2.7. Flow Cytometry

Isolated single mononuclear cells were obtained from the piglet PBMCs by density centrifugation using a porcine peripheral blood lymphocyte separation kit (Solarbio, Dalian, China). PBMCs were stained with anti-CD3-APC (BD Biosciences, San Diego, CA, USA), anti-CD4-FITC (BD Biosciences, San Diego, CA, USA), and anti-CD8-PE (BD Biosciences, San Diego, CA, USA) (1:100 dilution) for 30 min at 4 °C in the dark. The cells were then washed twice with PBS, and the expression of surface markers was observed using flow cytometry. The flow cytometry data were analyzed using the FlowJo software. A total of 10,000 lymphocytes were acquired per sample.

### 2.8. Statistical Analysis

Analysis of variance and unpaired Student’s *t*-tests were employed to determine statistically significant differences among multiple groups. The differences were considered significant at * *p* < 0.05, ** *p* < 0.01. Results are expressed as mean ± SEM.

## 3. Results

### 3.1. Histological Differences between Neonatal and Weaned Piglet Intestines

**Figure 1:**
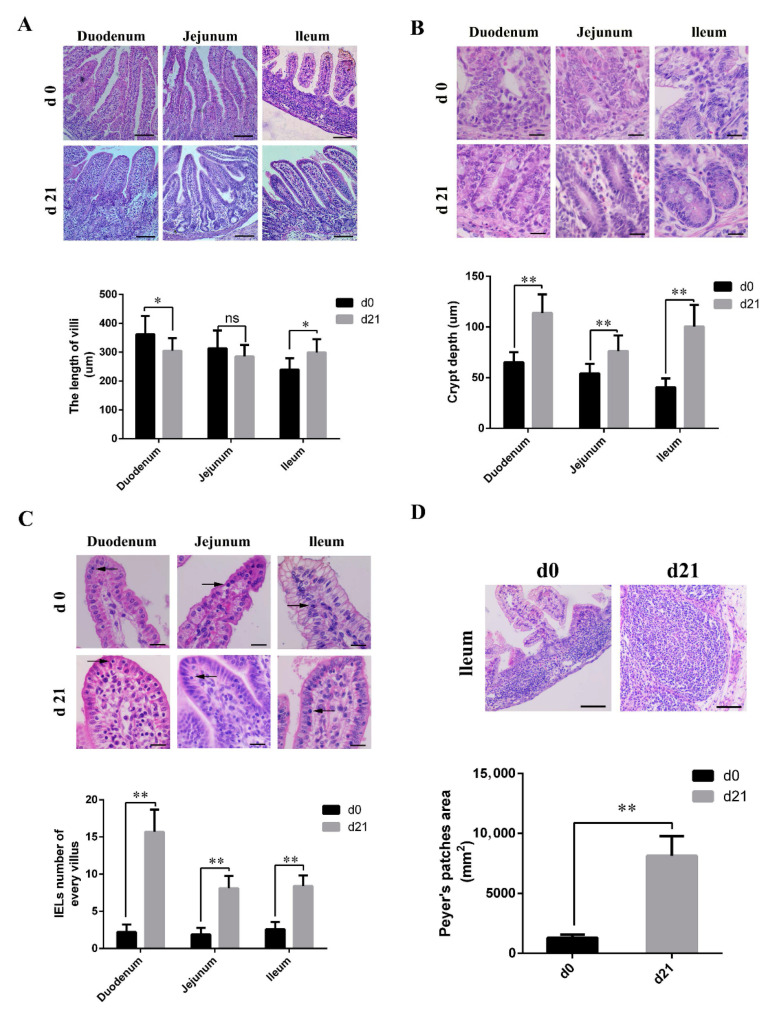
Histological and morphometrical differences between the small intestines of neonatal and weaned piglets. Tissue sections were stained using hematoxylin and eosin and certain parameters were compared between neonatal and weaned piglets. (**A**) Intestinal villus height. Scale bars: 100 μm. (**B**) Intestinal crypt depth. Scale bars: 20 μm. (**C**) Intestinal intraepithelial lymphocytes (→). Scale bars: 20 μm. (**D**) Ileal Peyer’s patches. Scale bars: 100 μm. The certain parameters were calculated in ten random fields. (*n* = 10 per group). * 0.01 < *p* < 0.05 and ** *p* < 0.01.

As shown in Figure 1A, microvilli in the small intestine of neonatal piglets appeared different from those of weaned piglets. Weaned piglets showed short, club, and blunt duodenal villi, and had longer ileal villi than those of the neonatal piglets. The length of the jejunal villi was similar for both neonatal and weaned piglets. In the weaned piglets, both the crypt depth (Figure 1B) and number of IELs (Figure 1C) were significantly higher than those in neonates, and had significantly larger and more mature PPs; moreover, the boundaries between the partial PPs were obscure in neonates (Figure 1D).

### 3.2. Weaners Have Superior Intestinal Barrier Function than Neonates

**Figure 2:**
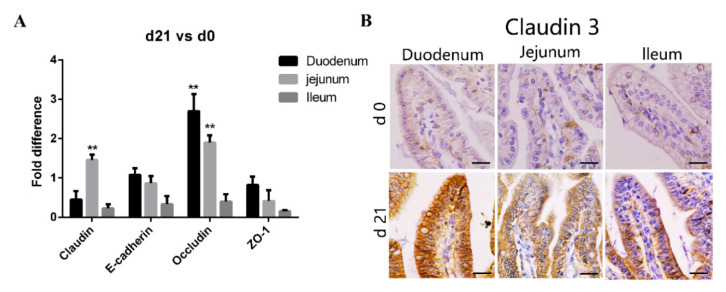
Differences in intestinal barrier function between neonatal and weaned piglets. (**A**) mRNA levels of claudin, E-cadherin, occludin, and ZO-1 in pig small intestine. All samples were tested in triplicate and the results are expressed as fold changes relative to the control animals. Data are presented as means ± standard errors. (**B**) Immunohistochemical staining of claudin-3 positive cells positive cell, Positive cells were quantified using densitometry analyses and were calculated in ten random fields. (*n* = 10 per group). Scale bars: 20 μm. ** *p* < 0.01.

Tight junctions, important determinants of epithelial barrier functions, are regulated by occludin, claudin, E-cadherin, and the zonula occludin (ZO) family [26]. Consequently, we compared the expression of genes coding for tight junction proteins in various areas of the small intestine between the neonatal and weaned piglets. As shown in Figure 2A, compared with that in neonates, the expression of claudin and occludin transcripts in the intestinal mucosa was significantly higher in weaners, while that of E-cadherin and ZO-1 was similar. We stained tissue sections with anti-claudin-3 antibodies to further compare the expression of tight junction proteins in various gut areas between the neonates and weaners and found that the expression of claudin-3 proteins was higher in weaners than in neonates, especially in the duodenum (Figure 2B).

### 3.3. Characteristics of Mucosal Immunity in Neonatal and Weaned Piglet Intestines

**Figure 3:**
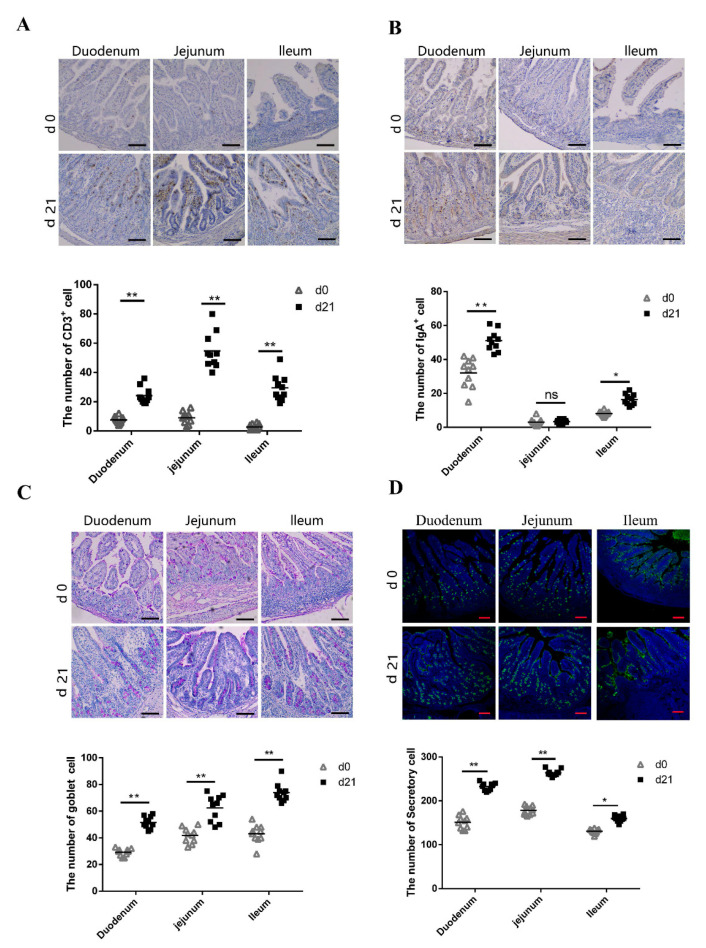
Expression of CD3^+^ T cells, SIgA-positive cells, goblet cells, and secretory cells in small intestines. (**A**–**C**) Immunohistochemical staining of CD3^+^ T cells, SIgA-positive cells, and goblet cells. ns: not significant. (**D**) Immunofluorescent staining of secretory cells in pig small intestine. Positive cells were quantified using densitometry analyses and were calculated in ten random fields. (*n* = 10 per group). Scale bars: 100 μm. * 0.01 < *p* < 0.05 and ** *p* < 0.01.

CD3^+^ T lymphocytes are the major T lymphocyte subtype [27,28]; hence, the number of CD3^+^ T lymphocytes could be considered as a proxy of the absolute T lymphocyte count. We compared the number of CD3^+^ T lymphocytes in the small intestine between neonatal and weaned piglets. We compared the number of CD3^+^ T lymphocytes in the small intestine between neonatal and weaned piglets and examined the CD3^+^ T lymphocyte distribution patterns using immunohistochemistry; immuno-positive cells were stained brown. Weaned piglets had more CD3^+^ T lymphocytes in the duodenum, jejunum, and ileum compared to neonatal piglets (Figure 3A). SIgA, the primary immunoglobulin isotype in animals, is primarily secreted across the intestinal mucosal surface, especially in the small intestine [29], plays an important role in intestinal mucosal immunity and is an index of intestinal mucosal immunity [30]. The number of IgA^+^ B cells on the small intestine mucosal surface were evaluated using immunohistochemistry; immuno-positive cells were stained brown. Weaned piglets had more IgA^+^ B cells in the duodenum and ileum than neonatal piglets, although there was no significant difference in the IgA^+^ B cell number between neonates and weaners (Figure 3B). Goblet cells (GCs) secrete anti-microbial proteins, chemokines, and cytokines and have important innate immunity-related functions besides intestinal barrier maintenance [31]. Herein, PAS-stained intestinal sections showed the presence of GCs in the intestinal mucosal surface epithelia of neonatal and weaned piglets. These cells, which appeared purple under PAS staining, are typically circular and cup-shaped and located in the intestinal lamina propria. As shown in Figure 3C, weaned piglets had more GCs in the duodenum, jejunum, and ileum than neonatal piglets. UEA-1, a universal marker of secretory cells, was also evaluated to determine the activation status of the intestinal cells [32]. Results showed that weaners had a higher proportion of intestinal secretory cells than neonates (Figure 3D).

### 3.4. Differences in Pattern Recognition Receptor Genes in the Intestinal Mucosa of Neonatal and Weaned Piglets

**Figure 4:**
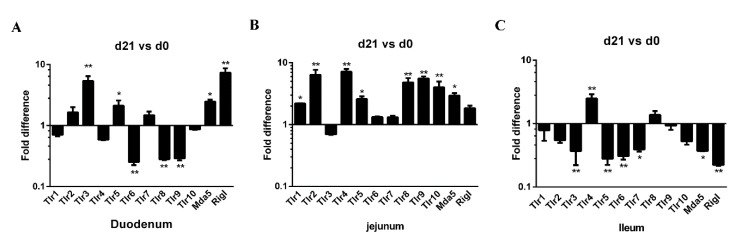
Differences in mRNA expression of PRR genes in neonatal and weaned piglet small intestines. (**A**–**C**) The mRNA levels of TLR1, TLR2, TLR 3, TLR4, TLR5, TLR6, TLR7, TLR8, TLR9, TLR10, MDA5, and Rig-I in the intestinal mucosa was determined in each animal using a SYBR-green qRT-PCR. All samples were tested in triplicate and the results are expressed as fold changes relative to the control animals. * 0.01 < *p* < 0.05 and ** *p* < 0.01.

Pattern recognition receptors (PRRs) are expressed on various mucosal cell types, such us Toll-like receptors (TLRs) and RIG-I-like receptors (RLRs), are important sensors in host-pathogen crosstalk. PRRs play a critical role in the detection of pathogen and in the elicitation of inflammatory and immune responses [33]. The expression of 12 PRR genes (TLR1–10, MDA5, and RIG-I) along the small intestine (duodenum, jejunum, and ileum) was examined in both neonatal and weaned piglets. In the duodenum, the expression of TLR3, TLR5, MDA5, and RIG-I genes was higher in weaners than in neonates, whereas duodenal TLR6, TLR8, and TLR9 expression was lower (Figure 4A). In the jejunum, the expression of TLR1, 2, 4, 5, 10, and MDA5 was higher in weaners than in neonates (Figure 4B), whereas in the ileum, only the expression of TLR 4 was high; while the expressions of TLR3, 5, 6, 7, MDA5, and RIG-I were lower in weaners than in neonates (Figure 4C).

### 3.5. Expression of Cytokines in the Intestinal Mucosa of Neonatal and Weaned Piglets

**Figure 5:**
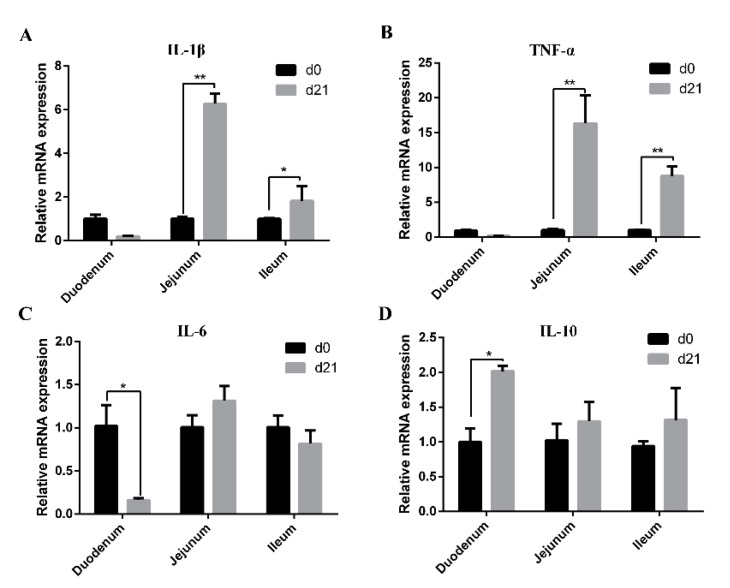
Expression of cytokines in the intestinal mucosa of neonatal and weaned piglets. (**A**–**D**) The mRNA expression levels of IL-1β, TNF-α, IL-6, and IL-10 in the intestinal mucosa was determined in each animal using a SYBR-green qRT-PCR. * 0.01 < *p* < 0.05 and ** *p* < 0.01.

The mRNA expression levels of IL-1β, TNF-α, IL-6, and IL-10 in the intestinal mucosa of neonatal and weaned piglets are shown in Figure 5. Compared with those in neonatal piglets, mRNA levels of IL-1β and TNF-α in jejunal and ileal mucosa as well as IL-10 in the duodenal mucosa were higher in weaned piglets (Figure 5A,B,D), while the mRNA level of IL-6 in duodenal mucosa was significantly decreased (Figure 5C).

### 3.6. Percentage of CD3^+^, CD4^+^, and CD8^+^ T Lymphocytes in Peripheral Blood Mononuclear Cells

**Figure 6:**
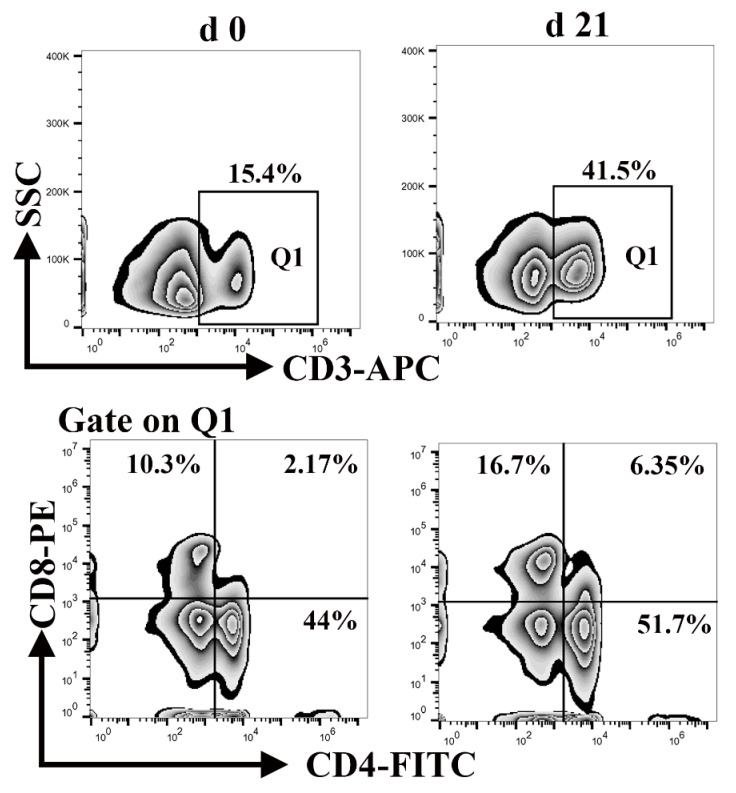
Differences in the number of CD3^+^, CD4^+^, and CD8^+^ T cells between neonatal and weaned piglets. The frequencies of CD3^+^, CD4^+^, and CD8^+^ T cells in the peripheral blood-mononuclear cells of neonatal and weaned piglets were analyzed using flow cytometry.

Lymphocytes, a major component of the peripheral innate immune system, are responsible for engulfing and killing pathogens during an infection [34]. Neonatal lymphocytes have quantitative deficiencies. As shown in Figure 6, the percentage of CD3^+^ T cells in the blood of neonatal and weaned piglets was 15.4% and 41.5%, respectively, whereas the percentage of CD4^+^ CD8^+^ T cells was 2.17% and 6.35%, respectively. This illustrates that the percentage of lymphocytes was significantly lower at birth than weaning. These results suggest that neonatal piglets are more susceptible to contracting infection.

## 4. Discussion

In animal production, enteric diarrhea is one of the most frequent early clinical signs of disease outbreak, with high morbidity and mortality [13,35]. Various enteric pathogens can cause intestinal diseases in piglets, which attributes to significant economic losses [8]. Neonatal piglets are highly susceptible to infection with viral and/or bacterial pathogens [9]. In this study, we compared the small intestinal morphology, immune cell composition, and expression of tight junction protein and PRRs gene in neonatal and weaned piglets to investigate the causes of susceptibility of neonatal piglets to infection and provide preliminary data for future mechanics and developmental studies.

Villus height, crypt depth, and epithelial barrier functions are indicators of gut health status in piglets and commonly used for studying intestinal morphology [36]. Our study showed that the shape, length, and other characteristics of these villi differed significantly between neonates and weaners, which may be correlated with dietary patterns. Pigs have different dietary requirements at different stages of development; hence, pigs’ intestines may morphologically change with age to accommodate changes in diet.

Intestinal crypts have numerous stem cells, which help to maintain the integrity of the intestinal epithelium and protect against possible injury caused by passing food [37,38]. Our study showed that the intestinal crypt depth of weaned piglets was significantly higher than that of neonatal piglets, possibly to produce more stem cells. Villagómez et al. has shown that IELs significantly influence the maintenance of the mucosal barrier integrity and protect against infections [39]. The present study indicated that, compared with weaned piglets, the immune system in neonatal piglets is not fully developed, as demonstrated by the fewer numbers of immune cells, such as CD3^+^ T, IgA^+^, GCs, secretory cells found in the intestines of neonates.

Tight junctions are a cell adhesion apparatus that act as barriers and/or channels in spaces between adjacent epithelial cells [40]. The expression of tight junction proteins in the intestinal tract is age specific. For example, claudin is normally expressed in the human fetal small intestine, but not in the adult colon under homeostatic conditions [41]. We determined the expression of genes coding for major intestinal tight junction proteins in neonatal and weaned piglet small intestinal tissue and found no significant difference in ZO-1 and E-cadherin gene expressions. However, the expression of claudin and occludin genes were different in the jejunum, while that of claudin-3 also differed at the protein level. These results suggest that weaners have better intestinal epithelial integrity than neonates. In addition, the intestinal imperfection of neonatal piglets facilitates the absorption of nutrients from breast milk, but also increases the risk of infection by pathogenic microorganisms.

PRRs are either expressed on cell surfaces or associated with intracellular vesicles that specifically bind to pathogen-associated molecular patterns (PAMPs) [42,43]. RNA viruses, including PEDV, can interact with several PRRs in the intestinal mucosa, such as TLRs and RLRs [23], thereby inducing an innate immune response. When microbes breach physical barriers, such as the skin or mucosa, TLRs recognize their PAMPs and induce an immune response [44]. Compared with post-weaning, the immune system of newborns is less developed, which may be related to TLR expression [45]. In line with previous findings, we have found that the number of TLRs in the duodenum and jejunum of weaners was relatively higher than that of neonatal piglets; however, the ileum showed opposite results.

Cytokines participate in immune and inflammatory responses and regulate intestinal barrier integrity [46]. Several pro-inflammatory cytokines, including IL-1β, IL-6, and TNF-α, are essential for pathogen clearance; however, excessive inflammation has adverse effects on intestinal mucosal integrity and epithelial function [46,47]. Conversely, anti-inflammatory cytokines, such as IL-10, can balance the pro inflammatory signals, limit severe inflammatory responses, and protect intestinal barrier function [48]. It has been reported that the expression of inflammatory cytokines in the intestine were significantly upregulated post weaning [48,49]. Herein, we have found that, compared with neonatal piglets, the mRNA levels of IL-1β and TNF-α in jejunal and ileal mucosa were higher in weaned piglets indicating that intestinal inflammation could clean pathogens and may have been restored in all piglets.

Lymphocytes are a major subclass of white blood cells that enter the blood circulation and facilitate the detection and elimination of pathogens and the dissemination of immunologic memory [50]. Differences observed in peripheral blood lymphocyte subpopulations in children compared to adults have prompted similar studies in animals. From birth until weaning piglets, CD4 ^+^ naive T cells count remains higher than CD8 ^+^ T cells [51]. Our results are consistent with previous reports. Moreover, we found that the percentage of lymphocytes was significantly lower in neonates than weaners; therefore, we suggest that disease diagnosis should be based on the size rather than the percentage of lymphocyte subpopulations, according to appropriate age-matched reference values.

## 5. Conclusions

Our study, to the best of our knowledge, is the first to evaluate the differences in the structure and immune capabilities of the small intestine between neonatal and weaned piglets. Our findings suggest that the difference in the physical and immunological components of the small intestines between neonatal and weaned piglets may explain the susceptibility of neonatal piglets to infection. This study provides essential preliminary data on the immune mechanisms of the small intestine for future studies.

## Figures and Tables

**Table 1:** Information of antibodies.

Primary Antibody	Secondary Antibody
Name; Catalog Number; Supplier; Dilution	Name, Catalog Number; Supplier
Rabbit anti-pig CD3 antibody; ab 16669 Abcam: 1:200	SABC-POD (rabbit IgG) Kit SA1022: BOSTER
Goat anti-pig IgA antibody; A100-102P: Bethyl; 1:100	SABC-POD (goat IgG) Kit; SA1023 BOSTER
Rabbit anti-Claudin3 antibody; ab 15102; abcam; 1:100	SABC-POD (rabbit IgG) Kit SA1022: BOSTER
FITC-UEA-I: 9006: sioma: 1:100	
Alexa FluorR 647 Mouse Anti-Pig CD38 BB23-8E6-8C8: BD Biosciences: 1:100	
PE Mouse Anti-Pig CD8a: 76-2-11: BD Biosciences: 1:100	
FITC Mouse anti-Pig CD4a 74-12-4: BD Biosciences: 1:100	

**Table 2:** Oligonucleotide PCR primers.

Gene	Primers Sequence (5–3)	Orientation
Claudin	AAGCCAAGATCCTCTACTCC	Forward
	GTAGTCCTT′GCGGTCGTA	Reverse
E-cadherin	AAATGCTTAGCTGGTGGGGAC	Forward
	GCCTCCCATTGCTAACACCT	Reverse
Occludin	ATCAACAAAGGCAACTCT	Forward
	GCAGCAGCCAT′GTACTCT	Reverse
Zo-1	AGCCCGAGGGGTGTTT	Forward
	GGTGGGAGGATGCTGTTG	Reverse
TLR1	AGATTTCGTGCCACCCTATG	Forward
	CCTGGGGGATAAACAATGTG	Reverse
TLR2	GAGTCTGCCACAACTCAAAGA	Forward
	CAGAACTGACAACATGGGTAGAA	Reverse
TLR3	GAGCAGGAGTTTGCCTTGTC	Forward
	GGAGGTCATCGGGTATTTGA	Reverse
TLR4	TCATCCAGGAAGGTTTCCAC	Forward
	TGTCCTCCCACTCCAGGTAG	Reverse
TLR5	GGTCCCTGCCTCAGTATCAA	Forward
	GTTGAGAAACCAGCTTGACG	Reverse
TLR6	TCAAGCATTTGGACCTCTCA	Forward
	TTCCAAATCCAGAAGGATGC	Reverse
TLR7	TCTGCCCTGTGATGTCAGTC	Forward
	GCTGGTTTCCATCCAGGTAA	Reverse
TLR8	CTGGGATGCTTGGTTCATCT	Forward
	CATGAGGTTGTCGATGATGG	Reverse
TLR9	AGGGAGACCTCTATCTCCGC	Forward
	AAGTCCAGGGTTTCCAGCTT	Reverse
TLR10	GCCCAAGGATAGGCGTAAAT	Forward
	CTCGAGACCCTTCATTCAGC	Reverse
Mda5	CAAGCTTGGGGAACGATGATG	Forward
	TAGCTGGTGATGGGGTCCTC	Reverse
RIG-I	GAGCCCTTGTGGATGCTTTA	Forward
	GGGTCATCCCTATGTTCTGATTC	Reverse
β-actin	GGACTTCGAGCAGGAGATGG	Forward
	AGGAAGGAGGGCTGGAAGAG	Reverse
IL-1β	AGAGGGACATGGAGAAGCGA	Forward
	GCCCTCTGGGTATGGCTTT	Reverse
TNF-α	GCCCTTCCACCAACGTTTTC	Forward
	TCCCAGGTAGATGGGTTCGT	Reverse
IL-6	F:CCTCGGCAAAATCTCTGCAA	Forward
	TGAAACTCCACAAGACCGGT	Reverse
IL-10	TCTGAGAACAGCTGCATCCAC	Forward
	CGCCCATCTGGTCCTTCGTT	Reverse

## Data Availability

The datasets used and analyzed during the current study are available from the corresponding author on reasonable request.

## Abbreviations

PRR	Pattern recognition receptor
PEDV	Porcine epidemic diarrhea virus
TGEV	Transmissible gastroenteritis virus
RVA	Rotavirus A
PBS	phosphate-buffered saline
H&E	Hematoxylin and eosin
DAPI	4′,6-diamidino-2-phenylindole
PPs	Peyer’s patches
IELs	Intraepithelial lymphocytes
GC	Goblet cell
UEA-1	Ulex europaeous agglutinin-1
TLR	Toll-like receptor
RLR	RIG-I-like receptor
PAMP	Pathogen-associated molecular pattern

## References

[1] Taylorpickard J.A., Spring P. (2008). Gut Efficiency: The Key Ingredient in Pig and Poultry Production.

[2] Brandtzaeg P. (2009). Mucosal Immunity: Induction, Dissemination, and Effector Functions. Scand. J. Immunol..

[3] Pluske J.R., Taylorpickard J.A., Spring P. (2008). Gut development: Interactions between nutrition, gut health and immunity in young pigs. Gut Efficiency: The Key Ingredient in Pig and Poultry Production.

[4] Lallès J.-P., Boudry G., Favier C., Le Floc’H N., Luron I., Montagne L., Oswald I.P., Pié S., Piel C., Sève B. (2004). Gut function and dysfunction in young pigs: Physiology. Anim. Res..

[5] AbreuMartin M.T., Targan S.R. (1996). Regulation of immune responses of the intestinal mucosa. Crit. Rev. Immunol..

[6] Li Y., Wu Q.X., Huang L.L., Yuan C., Wang J.L., Yang Q. (2018). An alternative pathway of enteric PEDV dissemination from nasal cavity to intestinal mucosa in swine. Nat. Commun..

[7] Laude H., Rasschaert D., Delmas B., Godet M., Gelfi J., Charley B. (1990). Molecular biology of transmissible gastroenteritis virus. Vet. Microbiol..

[8] Katsuda K., Kohmoto M., Kawashima K., Tsunemitsu H. (2006). Frequency of enteropathogen detection in suckling and weaned pigs with diarrhea in Japan. J. Vet. Diagn..

[9] Mesonero-Escuredo S., Strutzberg-Minder K., Casanovas C., Segalés J. (2018). Viral and bacterial investigations on the aetiology of recurrent pig neonatal diarrhoea cases in Spain. Porc. Health Manag..

[10] Baxter E. (1998). Causes and mitigation strategies for mortality in neonatal and weaned piglets. J. Anim. Sci..

[11] Hampson D.J. (1986). Alterations in piglet small intestinal structure at weaning. Res. Vet. Sci..

[12] Basha S., Surendran N., Pichichero M. (2014). Immune responses in neonates. Expert Rev. Clin. Immunol..

[13] Holland R.E. (1990). Some infectious causes of diarrhea in young farm animals. Clin. Microbiol. Rev..

[14] Yang H., Xiong X., Wang X., Bie T., Li T., Yin Y., Han X. (2016). Effects of Weaning on Intestinal Upper Villus Epithelial Cells of Piglets. PLoS ONE.

[15] Xun W., Shi L., Zhou H., Hou G., Cao T. (2018). Effect of weaning age on intestinal mucosal morphology, permeability, gene expression of tight junction proteins, cytokines and secretory IgA in Wuzhishan mini piglets. Ital. J. Anim. Sci..

[16] Verdile N., Mirmahmoudi R., Brevini T.A.L., Gandolfi F. (2019). Evolution of pig intestinal stem cells from birth to weaning. Animal.

[17] Kararli T.T. (1995). Comparison of the Gastrointestinal Anatomy, Physiology, and Biochemistry of Humans and Commonly Used Laboratory-Animals. Biopharm. Drug Dispos..

[18] Nejdfors P., Ekelund M., Jeppsson B., Westrom B.R. (2000). Mucosal in vitro permeability in the intestinal tract of the pig, the rat, and man: Species- and region-related differences. Scand. J. Gastroenterol..

[19] Patterson J.K., Lei X.G., Miller D.D. (2008). The pig as an experimental model for elucidating the mechanisms governing dietary influence on mineral absorption. Exp. Biol. Med..

[20] Mou C., Zhu L., Xing X., Lin J., Yang Q. (2016). Immune Responses Induced by Recombinant *Bacillus Subtilis* Expressing the Spike Protein of Transmissible Gastroenteritis Virus in pigs. Antivir. Res..

[21] Zhang E., Wang J., Li Y., Huang L., Yang Q. (2020). Comparison of oral and nasal immunization with inactivated porcine epidemic diarrhea virus on intestinal immunity in piglets. Exp. Ther. Med..

[22] Zong Q.F., Huang Y.J., Wu L.S., Wu Z.C., Wu S.L., Bao W.B. (2019). Effects of porcine epidemic diarrhea virus infection on tight junction protein gene expression and morphology of the intestinal mucosa in pigs. Pol. J. Vet. Sci..

[23] Temeeyasen G., Sinha A., Gimenez-Lirola L.G., Zhang J.Q., Pi Eyro P.E. (2018). Differential gene modulation of pattern-recognition receptor TLR and RIG-I-like and downstream mediators on intestinal mucosa of pigs infected with PEDV non S-INDEL and PEDV S-INDEL strains. Virology.

[24] Lu X., Yunhan Y., Jialu W., Yuchao J., Qian Y. (2018). Impact of TGEV infection on the pig small intestine. Virol. J..

[25] Wang F., Wang S.Q., Wang H.F., Wu Z.C., Wu S.L. (2020). Effects of porcine epidemic diarrhea virus infection on Toll-like receptor expression and cytokine levels in porcine intestinal epithelial cells. Pol. J. Vet. Sci..

[26] Jung K., Eyerly B., Annamalai T., Lu Z., Saif L.J. (2015). Structural alteration of tight and adherens junctions in villous and crypt epithelium of the small and large intestine of conventional nursing piglets infected with porcine epidemic diarrhea virus. Vet. Microbiol..

[27] Haverson K., Bailey M., Stokes C.R. (1999). T-cell populations in the pig intestinal lamina propria: Memory cells with unusual phenotypic characteristics. Immunology.

[28] Kokuina E., Breff-Fonseca M.C., Villegas-Valverde C.A., Mora-Diaz I. (2019). Normal Values of T, B and NK Lymphocyte Subpopulations in Peripheral Blood of Healthy Cuban Adults. MEDICC Rev..

[29] Pabst O., Slack E. (2020). IgA and the intestinal microbiota: The importance of being specific. Mucosal Immunol..

[30] Mantis N.J., Rol N., Corthesy B. (2011). Secretory IgA’s complex roles in immunity and mucosal homeostasis in the gut. Mucosal Immunol..

[31] Knoop K.A., Newberry R.D. (2018). Goblet cells: Multifaceted players in immunity at mucosal surfaces. Mucosal Immunol..

[32] Gonzalez L.M., Williamson I., Piedrahita J.A., Blikslager A.T., Magness S.T. (2013). Cell Lineage Identification and Stem Cell Culture in a Porcine Model for the Study of Intestinal Epithelial Regeneration. PLoS ONE.

[33] Mogensen T.H. (2009). Pathogen recognition and inflammatory signaling in innate immune defenses. Clin. Microbiol. Rev..

[34] Nascimbeni M., Shin E.C., Chiriboga L., Kleiner D.E., Rehermann B. (2004). Peripheral CD4(+)CD8(+) T cells are differentiated effector memory cells with antiviral functions. Blood.

[35] Morin M., Turgeon D., Jolette J., Robinson Y., Larivière S. (1983). Neonatal diarrhea of pigs in Quebec: Infectious causes of significant outbreaks. Can. J. Comp. Med..

[36] Han F., Hu L., Xuan Y., Ding X., Luo Y., Bai S., He S., Zhang K., Che L. (2013). Effects of high nutrient intake on the growth performance, intestinal morphology and immune function of neonatal intra-uterine growth-retarded pigs. Br. J. Nutr..

[37] Sato T., Clevers H. (2013). Growing Self-Organizing Mini-Guts from a Single Intestinal Stem Cell: Mechanism and Applications. Science.

[38] Moossavi S., Zhang H., Sun J., Rezaei N. (2013). Host-microbiota interaction and intestinal stem cells in chronic inflammation and colorectal cancer. Expert Rev. Clin. Immunol..

[39] Olivares-Villagómez D., Kaer L.V. (2018). Intestinal Intraepithelial Lymphocytes: Sentinels of the Mucosal Barrier. Trends Immunol..

[40] Nakamura S., Irie K., Tanaka H., Nishikawa K., Suzuki H., Saitoh Y., Tamura A., Tsukita S., Fujiyoshi Y. (2019). Morphologic determinant of tight junctions revealed by claudin-3 structures. Nat. Commun..

[41] Luettig J., Rosenthal R., Barmeyer C., Schulzke J. (2015). Claudin-2 as a mediator of leaky gut barrier during intestinal inflammation. Tissue Barriers.

[42] Takeuchi O., Akira S. (2010). Pattern Recognition Receptors and Inflammation. Cell.

[43] Uematsu S., Akira S. (2008). Toll-Like receptors (TLRs) and their ligands. Handb. Exp. Pharmacol..

[44] Flaherty S., Reynolds J.M. (2016). TLR Function in Murine CD4(+) T Lymphocytes and Their Role in Inflammation. Methods. Mol. Biol..

[45] Bailey M., Haverson K., Inman C., Harris C., Jones P., Corfield G., Miller B., Stokes C. (2005). The development of the mucosal immune system pre- and post-weaning: Balancing regulatory and effector function. Proc. Nutr. Soc..

[46] Al-Sadi R. (2009). Mechanism of cytokine modulation of epithelial tight junction barrier. Front. Biosci..

[47] Liu Y., Huang J., Hou Y., Zhu H., Zhao S., Ding B., Yin Y., Yi G., Shi J., Fan W. (2008). Dietary arginine supplementation alleviates intestinal mucosal disruption induced by Escherichia coli lipopolysaccharide in weaned pigs. Br. J. Nutr..

[48] Madsen K.L., Lewis S.A., Tavernini M.M., Hibbard J., Fedorak R.N. (1997). Interleukin 10 prevents cytokine-induced disruption of T84 monolayer barrier integrity and limits chloride secretion. Gastroenterology.

[49] Hu C.H., Xiao K., Luan Z.S., Song J. (2013). Early weaning increases intestinal permeability, alters expression of cytokine and tight junction proteins, and activates mitogen-activated protein kinases in pigs. J. Anim. Sci..

[50] Andrade W.N., Johnston M.G., Hay J.B. (1998). The relationship of blood lymphocytes to the recirculating lymphocyte pool. Blood.

[51] Mccauley I., Hartmann P.E. (1984). Changes in the proportion and absolute number of T lymphocytes in piglets from birth until after weaning and in adults. Res. Vet. Sci..

